# Early and Short-Term Interventions in the Gut Microbiota Affects Lupus Severity, Progression, and Treatment in MRL/lpr Mice

**DOI:** 10.3389/fmicb.2020.00628

**Published:** 2020-04-14

**Authors:** Yun Zhang, Qiuping Liu, Yiran Yu, Mingzhu Wang, Chengping Wen, Zhixing He

**Affiliations:** Institute of Basic Research in Clinical Medicine, College of Basic Medical Science, Zhejiang Chinese Medical University, Hangzhou, China

**Keywords:** gut microbiota, lupus, antibiotics, fecal microbiota transplantation, prednisone

## Abstract

There have been attempts to reveal the possible associations between systemic lupus erythematosus (SLE) and gut microbiota. Using MRL/lpr mice, this study was performed to reveal whether early and short-term interventions in gut microbiota affect lupus. MRL/lpr mice were treated with antibiotics or fecal microbiota transplantation (FMT) before onset. Then, prednisone was used to treat the lupus mice with initially different gut microbiota compositions. The compositions of gut microbiota were assessed by the V3-V4 region of 16S rRNA gene sequence. Early and short-term antibiotics exposure aggravated lupus severity by depleting beneficial gut microbiota for lupus, such as *Lactobacillus* and *Bifidobacterium*, and enriching harmful gut microbiota for lupus, such as *Klebsiella* and *Proteus*. FMT alleviated lupus severity by renovating the antibiotic-induced dysbiosis of gut microbiota in the following 1 week after antibiotics exposure. Besides, short-term antibiotics exposure before onset imposed no significant effects on lupus progression, but the following one week of FMT suppressed lupus progression. Moreover, the short-term antibiotics or FMT before onset inhibited the therapeutic efficiency of prednisone on lupus from 9 to 13 weeks old of MRL/lpr mice. These data demonstrate that the gut microbiota before onset is important for lupus severity, progression and treatment.

## Introduction

Systemic lupus erythematosus (SLE) is a systemic autoimmune disease characterized by lymphocyte over activation and autoantibody production (Wen et al., 2016). The pathogenesis of SLE is influenced by a combination of genetic and environmental factors. Genetic studies of individuals with SLE have identified multiple risked loci, but fail to predict with sufficient precision those who will ultimately develop the disease (Jeong et al., 2018). Increasing evidence has indicated the importance of environmental triggers for SLE pathogenesis (Gulati and Brunner, 2018). The gut microbiota, plays major roles in antibody production, shaping the human B cell repertoire and maintaining the homeostasis of different populations of helper T cells and the Th17: Treg balance, is one such environmental factor correlated with SLE disease manifestations (Lopez et al., 2016). The disturbance of the gut microbiota, called dysbiosis, has been demonstrated in SLE patients and lupus mice models (Hevia et al., 2014; Zhang et al., 2014; Luo et al., 2018). Furthermore, the modification of the gut microbiota can affect lupus activity (Mu et al., 2017a; Ma et al., 2019). However, whether dysbiosis is merely a consequence of lupus progressions or is itself pathogenic remains unknown.

Antibiotic-induced dysbiosis is a common method for studying the role of gut microbiota in the pathogenesis of disease (Ianiro et al., 2016). For instance, intestinal dysbiosis triggers mucosal immune responses that stimulate T and B cells, which are key to the development of rheumatoid arthritis (Jubair et al., 2018). Recently, another study has also described that antibiotics ameliorated lupus disease by changing the composition of the gut microbiota in the classical SLE mouse model MRL/Mp-Fas^lpr^ (MRL/lpr) (Mu et al., 2017a). The antibiotic mixture was given in the drinking water after disease onset from 9 weeks of age until euthanasia at 16 weeks of age (Mu et al., 2017a). However, questions from this study are whether changes in the gut microbiota occurred after the onset of lupus disease and whether these changes are a consequence of antibiotics ameliorating lupus disease or are its cause. Furthermore, this study did not address the effects of the gut microbiota composition on lupus treatment.

Given the outstanding questions regarding the effects of changing gut microbiota before disease onset on lupus disease, antibiotics were used to induce intestinal dysbiosis from 6 weeks of age until 8 weeks of age in MRL/lpr mice. Then, fecal microbiota from C57/BL6 mice was transplanted into antibiotic-treated MRL/lpr mice by fecal microbiota transplantation (FMT). Lupus activity was determined at 9, 11, and 13 weeks old in three types of MRL/lpr mice with different initial gut microbiota compositions. In addition, the efficacy of prednisone was evaluated in the three types of MRL/lpr mice.

## Materials and Methods

### Mice and Housing

Specific pathogen free (SPF) grade female MRL/lpr mice (6 weeks old) and C57/BL6 mice (6 weeks old) were purchased from Shanghai SLAC Laboratory Animal Co., Ltd. (Shanghai, China) and maintained in the SPF environment of the Zhejiang Chinese Medical University Laboratory Animal Research Center. The mice were housed in controlled environments with free access to food and water. The mice were weighed twice weekly, and the drug doses were adjusted accordingly. All animal experiments were performed according to the requirements of the Institutional Animal Care and Use Committee of China.

### Study Design and Treatment Administration

**FIGURE 1:**
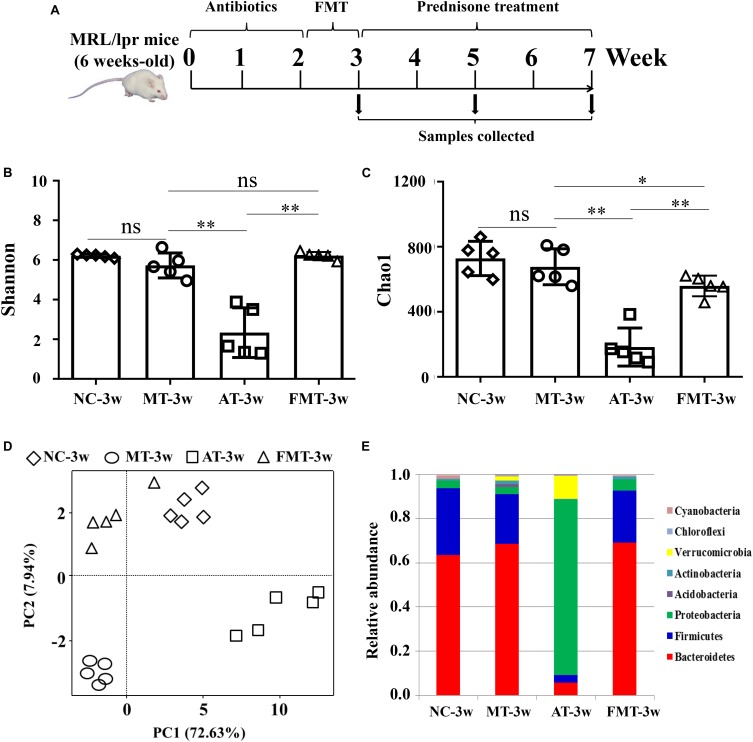
Alterations in gut microbiota caused by antibiotics or fecal microbiota transplantation. **(A)** A diagram of the experimental treatments is shown. **(B)** The Shannon index of the gut microbiota is shown. **(C)** The Chao1 index of the gut microbiota is shown. **(D)** Alterations in the overall community structures are shown in the PCoA plot. **(E)** The percent abundance of the identified major phylum is shown. NC, The C57/BL6 mice treated with PBS solution from 6 to 9 weeks old; MT, the MRL/lpr mice treated with PBS solution from 6 to 9 weeks old; AT, the MRL/lpr mice treated with antibiotics from 6 to 8 weeks old and PBS solution from 8 to 9 weeks old; FMT, the MRL/lpr mice treated with antibiotics from 6 to 8 weeks old and fecal microbiota transplantation from 8 to 9 weeks old. “^∗∗^” represents *p* < 0.01; “^∗^” represents *p* < 0.05; “ns” represents no significant difference. *N* = 5/group.

The time course and grouping information for the treatments is shown in Figure 1A. In the first 2 weeks, the mice were treated with a cocktail of broad-spectrum antibiotics, including ampicillin (0.2 g/L), vancomycin (0.1g/L), neomycin (0.2 g/L), and metronidazole (0.2 g/L), in their drinking water. A previous study reported that 2 weeks of antibiotics leads to intestinal dysbiosis (Aguilera et al., 2015). Then, a portion of the mice was orally gavaged with a 200 μL/day aliquot of fecal suspensions (initial dilution 5 g/mL feces) for 1 week. The fecal suspensions were prepared by resuspending the fresh feces of normal C57/BL6 (8 weeks old) in phosphate-buffered saline (PBS) as previously described (Ekmekciu et al., 2017). After the intervention of gut microbiota, the MRL/lpr mice with different types of gut microbiota were orally gavaged with 5 mg/kg prednisone every day for 4 weeks. The mice that did not receive FMT or prednisone treatments were subjected to oral gavages of water to match the stress of gavage manipulation every day. The entire experimental period was 7 weeks, starting 1 day after antibiotic administration. The sampling times were the 3rd, 5th, and 7th weeks. Each group contains five mice.

### Microbiome Analysis

Colonic microbiota samples were collected within 10 min after euthanization. To avoid cross-contamination, each microbiota sample was collected by using a new pair of sterile tweezers. Total genomic DNA was extracted from each stool sample using a QIAamp^®^ DNA Stool Mini Kit (Qiagen, Hilden, Germany) according to the manufacturer’s protocols. Harvested DNA was PCR amplified with broad-range bacterial primers targeting the V3-V4 region of the 16S rRNA gene as previously described (Yu et al., 2018). Subsequently, the amplicons were purified according to standard procedures, quantified, pooled and sequenced with the MiSeq Reagents Kit v3 (600 cycles, Illumina) according to the manufacturer’s instructions with 20% PhiX (Illumina). The sequencing reaction was conducted by Hangzhou Guhe Information and Technology Co., Ltd., Zhejiang, China.

The processing and quality filtering of the reads were performed by using scripts in Quantitative Insights into Microbial Ecology (QIIME, Version 1.9) (Caporaso et al., 2010). The clean reads were extracted from the raw-paired end reads according to previous studies (Yu et al., 2018). UCLUST was used to cluster sequencing reads into operational taxonomical units (OTUs based on > 97% identity) (Edgar, 2010). Bacterial taxonomy was assigned by using the SILVA (Quast et al., 2013) and NCBI databases (Sayers et al., 2019). The microbiota OTUs were imported into R version 3.4.3 and alpha and beta diversity metrics were computed using the “vegan” package. Alpha diversity included the Shannon and Chao1 diversity index, and beta diversity included unweighted UniFrac distance metrics. Beta diversity metrics were then visualized using Principal coordinate analyses (PCoA) in R version 3.4.3. OTUs with > 0.05% mean abundance in one sample and observed in > 10% of the samples were included in differential analyses. The linear discriminant analysis (LDA) effect size (LEfSe) method was carried out using Galaxy^1^, with a set logarithmic LDA score of 2.0 and the standard test for the significant difference between the two groups (Kruskal–Wallis test and Wilcoxon rank-sum Test) (Segata et al., 2011).

### Biochemical Measurements

Serum creatinine (Cr) and alanine aminotransferase (ALT) were measured via an enzymatic-colorimetric method using standard test kits on a TBA-40FR automated biochemical analyzer (Toshiba, Japan). Serum lactate dehydrogenase (LDH) activity was determined using the lactate dehydrogenase assay kit (Jiancheng, China) according to the manufacturer’s instructions. A multi-analyte ELISA was used to measure the levels of anti-dsDNA antibody (Shibayagi, Japan), IFN-α (Invitrogen, United States) and IL-6 (Invitrogen, United States) in serum according to the manufacturer’s instructions.

### Statistical Analysis

All results were presented as Mean ± SEM of data from at least three independent experiments. Differences between groups were evaluated with one-way ANOVA. Statistical analysis was performed using Prism 5.0 (GraphPad Software, United States). A significance level of *p* < 0.05 was considered statistically significant.

## Results

### Lupus Severity Was Affected by Early and Short-Term Antibiotics Exposure and FMT

**FIGURE 2:**
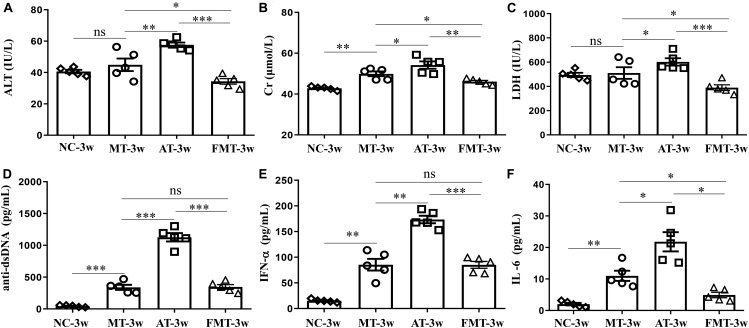
Serum levels of alanine aminotransferase activity **(A)**, creatinine **(B)**, lactate dehydrogenase activity **(C)**, anti-double stranded DNA antibody **(D)**, interferon-α **(E)** and interleukin-6 **(F)** in MRL/lpr mice from the three groups. NC, The C57/BL6 mice treated with PBS solution from 6 to 9 weeks old; MT, the MRL/lpr mice treated with PBS solution from 6 to 9 weeks old; AT, the MRL/lpr mice treated with antibiotics from 6 to 8 weeks old and PBS solution from 8 to 9 weeks old; FMT: the MRL/lpr mice treated with antibiotics from 6 to 8 weeks old and fecal microbiota transplantation from 8 to 9 weeks old. “^∗∗∗^”represents *p* < 0.001; “^∗∗^” represents *p* < 0.01; “^∗^” represents *p* < 0.05; “ns” represents no significant difference. Green arrows indicate the glomerulus. *N* = 5/group.

At 9 weeks-old of age, there were some differences in gut microbiota between MRL/lpr and C57/BL6 mice (Figures 1D,E, Supplementary Figure S1A, and Table 1). However, the MRL/lpr mice had significantly higher levels of autoantibodies and inflammatory factors than the C57/BL6 mice (Figure 2). After short-term antibiotics exposure, the alpha diversity of gut microbiota significantly reduced (Figures 1B,C) and the overall compositions of gut microbiota significantly changed (Figure 1D). At the phylum level, antibiotics caused significant decreases in Firmicutes and Bacteroidetes but significant increases in Proteobacteria and Verrucomicrobia (Figure 1E). At the genus level, antibiotics significantly downregulated 17 genera, including *Bifidobacterium*, *Bacteroides*, and *Lactobacillus*, and only two genera (*Klebsiella* and *Proteus*) were upregulated by antibiotics (Supplementary Figure S1B and Table 1). To renovate intestinal dysbiosis, the fecal microbiota from normal C57BL/6J mice was transplanted into the antibiotic exposure mice in the following 1 week. The alpha diversity and abundances of Firmicutes and Bacteroidetes were resorted after FMT (Figures 1B,C,E). Additionally, FMT could resort to the abundance of 10 genera changed by antibiotics, such as *Bifidobacterium*, *Adlercreutzia*, *Bacteroides*, *Klebsiella*, and *Proteus* (Supplementary Figure S1C and Table 1). However, the gut microbiota in FMT-treated mice was also inconsistent with that in the model mice (Figure 1D). Compared with the model mice, the abundance of three genera was higher, and the abundance of seven genera was lower in the FMT-treated mice (Supplementary Figure S1D and Table 1). In sum, this study successfully obtained three types of lupus mice with differently initial gut microbiota compositions before onset.

**TABLE 1:** Significantly different genus among the four types of mice at 9 weeks old.

	NC vs. MT		MT vs. AT		MT vs. FMT		FMT vs. AT	
	NC	MT	MT	AT	MT	FMT	FMT	AT
*Bifidobacterium*			↑				↑	
*Adlercreutzia*	↑		↑				↑	
*Bacteroides*		↑	↑		↑		↑	
*Parabacteroides*		↑	↑		↑			
*Lactobacillus*			↑				↑	
*Turicibacter*			↑				↑	
*Dehalobacterium*			↑				↑	
*(Ruminococcus)*		↑	↑		↑		↑	
*Coprococcus*		↑	↑		↑		↑	
*Dorea*		↑	↑		↑			
*Oscillospira*			↑				↑	
*Ruminococcus*			↑				↑	
*Allobaculum*	↑		↑				↑	
*Sutterella*			↑			↑	↑	
*Bilophila*		↑	↑		↑		↑	
*Klebsiella*				↑				↑
*Proteus*				↑				↑
*Odoribacter*						↑	↑	
*Desulfovibrio*	↑					↑	↑	
*Akkermansia*		↑			↑			↑
*Prevotella*	↑						↑	

**“↑” represent significantly higher in comparisons. *NC, The C57/BL6 mice treated with PBS solution from 6 to 9 weeks old; MT, the MRL/lpr mice treated with PBS solution from 6 to 9 weeks old; AT, the MRL/lpr mice treated with antibiotics from 6 to 8 weeks old and PBS solution from 8 to 9 weeks old; FMT, the MRL/lpr mice treated with antibiotics from 6 to 8 weeks old and fecal microbiota transplantation from 8 to 9 weeks old.***

After the intervention of gut microbiota before onset, lupus activity showed being different in three types of MRL/lpr mice. As shown in Figure 2, the levels of 3 serum biochemical indexes important for liver and kidney function (ALT, Cr, and LDH) were significantly increased by the early and short-term antibiotics exposure. Although FMT could significantly alleviate the liver and kidney damage caused by antibiotics exposure, it did not eliminate the influence of antibiotics exposure (Figure 2). Anti-dsDNA autoantibodies are the most studied antibodies with lupus-related autoantibodies. The early and short-term antibiotics exposure caused significant increases in serum anti-dsDNA autoantibodies in MRL/lpr mice, and FMT reduced serum anti-dsDNA autoantibodies to levels before antibiotic exposure (Figure 2D). The effects of antibiotics and FMT on anti-dsDNA autoantibodies were consistent with their effects on inflammatory cytokines (IFN-α and IL-6) (Figures 2E,F).

### Lupus Progression Was Not Affected by Short-Term Antibiotics Exposure but Suppressed by Short-Term FMT Treatment

**FIGURE 3:**
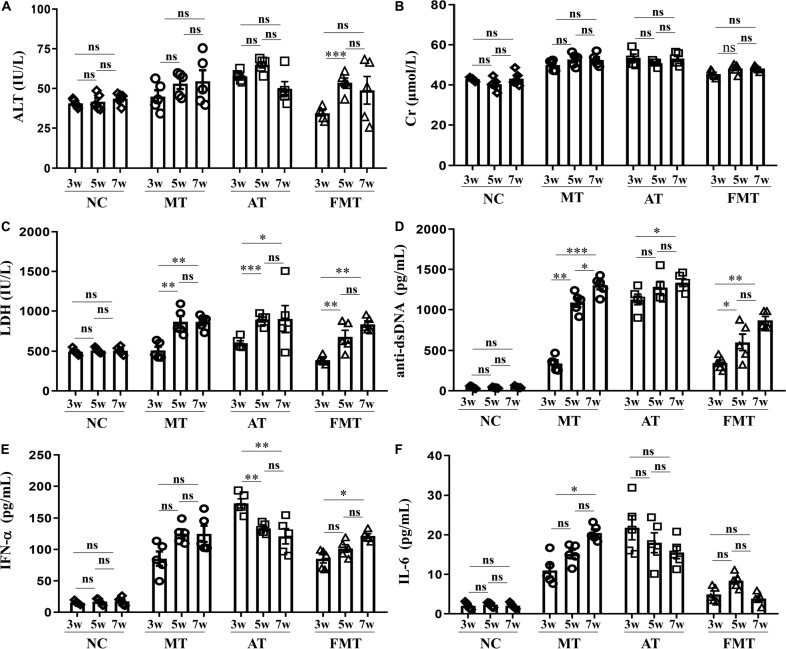
Dynamics of serum alanine aminotransferase activity **(A)**, creatinine **(B)**, lactate dehydrogenase activity **(C)**, anti-double stranded DNA antibody **(D)**, interferon-α **(E)** and interleukin-6 **(F)** from 9 to 13 weeks old in MRL/lpr mice from three groups. NC, The C57/BL6 mice treated with PBS solution from 6 to 9 weeks old; MT, the MRL/lpr mice treated with PBS solution from 6 to 9 weeks old; AT: the MRL/lpr mice treated with antibiotics from 6 to 8 weeks old and PBS solution from 8to 9 weeks old; FMT, the MRL/lpr mice treated with antibiotics from 6 to 8 weeks old and fecal microbiota transplantation from 8 to 9 weeks old. “^∗∗∗^”represents *p* < 0.001; “^∗∗^” represents *p* < 0.01; “^∗^” represents *p* < 0.05; “ns” represents no significant difference. *N* = 5/group.

In addition, this study also revealed the effects of the early and short-term antibiotics exposure and FMT on lupus progression. There were no differences in the developments of ALT and Cr, but some differences in the development of LDH, anti-dsDNA, IFN-α, and IL-6 were observed among the three groups (Figure 3). The levels of LDH and anti-dsDNA autoantibody were significantly increased from 9 to 13 weeks old in MRL/lpr mice of all groups. In addition, the anti-dsDNA levels in FMT-treated mice were the lowest among the three types of mice at 11 and 13 weeks old (Figure 3D). Interestingly, the level of IFN-α or IL-6 showed different development with anti-dsDNA among the three groups (Figures 3E,F). In the control mice, both IFN-α and IL-6 had significantly increased development from 9 to 13 weeks old. In the antibiotic-treated mice, IFN-α showed a significant decrease, and IL-6 showed an unobvious decrease from 9 to 13 weeks old (Figures 3E,F). In the FMT-treated mice, IFN-α showed a significant increase, but IL-6 showed an unobvious alteration from 9 to 13 weeks old (Figures 3E,F). In sum, early and short-term antibiotics exposure imposed no significant effects on lupus progression, but the following 1 week of FMT suppressed lupus progression in MRL/lpr mice from 9 to 13 weeks old.

### Prednisone Efficiency on Lupus Was Inhibited by Early and Short-Term Antibiotics Exposure and FMT

**FIGURE 4:**
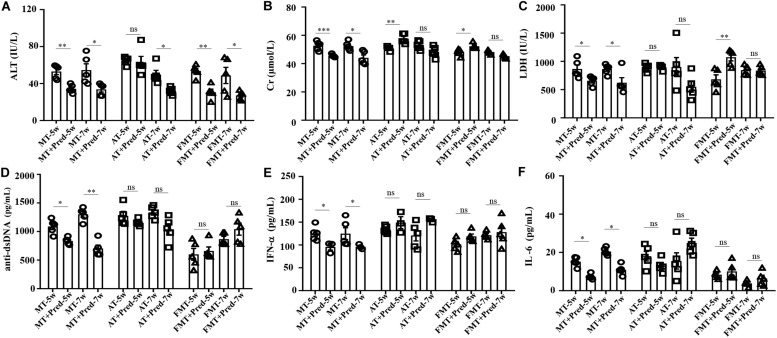
Prednisone-induced alterations in serum levels of alanine aminotransferase activity **(A)**, creatinine **(B)**, lactate dehydrogenase activity **(C)**, anti-double stranded DNA antibody **(D)**, interferon-α **(E)** and interleukin-6 **(F)** in MRL/lpr mice from three groups. NC, The C57/BL6 mice treated with PBS solution from 6 to 9 weeks old; MT, the MRL/lpr mice treated with PBS solution from 6 to 9 weeks old; AT, the MRL/lpr mice treated with antibiotics from 6 to 8 weeks old and PBS solution from 8 to 9 weeks old; FMT, the MRL/lpr mice treated with antibiotics from 6 to 8 weeks old and fecal microbiota transplantation from 8 to 9 weeks old. “^∗∗∗^” represents *p* < 0.001; “^∗∗^” represents *p* < 0.01; “^∗^” represents *p* < 0.05; “ns” represents no significant difference. Pred, prednisone. *N* = 5/group.

In our previous study, interventions in the gut microbiota could affect the efficacy of prednisone on lupus (He et al., 2019). This study also demonstrated that the efficacy of prednisone was influenced by early and short-term intervention in gut microbiota by antibiotics exposure and FMT. In the control mice, prednisone showed significant therapeutic effects on lupus according to the decreases in ALT, Cr, LDH, anti-dsDNA, IFN-α, and IL-6 (Figure 4). However, prednisone could not alleviate lupus in both antibiotic-treated and FMT-treated mice (Figure 4). The level of anti-dsDNA autoantibody was reduced by prednisone in the early and short-term antibiotic exposure mice, but this effect was not statistically significant (Figure 4D). Similarly, the levels of IFN-α and IL-6 were not significantly influenced by prednisone in the early and short-term antibiotic exposure mice (Figures 4E,F). In the early and short-term FMT-treated mice, prednisone also caused no significant alteration in anti-dsDNA autoantibodies, IFN-α, and IL-6 (Figures 4D–F).

**FIGURE 5:**
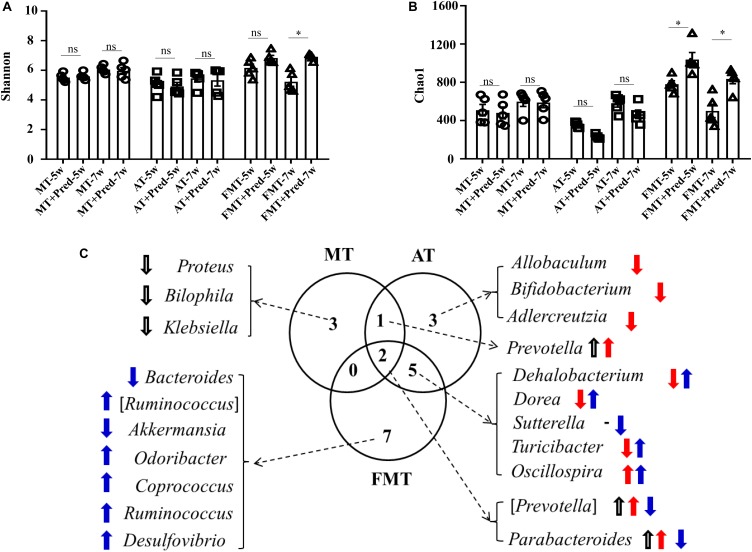
Prednisone-induced alterations in alpha diversity [Shannon **(A)** and Chao1 **(B)**] in the three types of MRL/lpr mice. Venn diagrams **(C)** show the distribution of shared and unique prednisone-induced microbial genera in the MT, AT, and FMT groups. NC, The C57/BL6 mice treated with PBS solution from 6 to 9 weeks old; MT, the MRL/lpr mice treated with PBS solution from 6 to 9 weeks old; AT: the MRL/lpr mice treated with antibiotics from 6 to 8 weeks old and PBS solution from 8 to 9 weeks old; FMT, the MRL/lpr mice treated with antibiotics from 6 to 8 weeks old and fecal microbiota transplantation from 8 to 9 weeks old. “*” represents *p* < 0.05; “ns” represents no significant difference. Black, red, and blue arrows represent the alterations in control, antibiotic-treated and FMT treated mice, respectively. Up arrow represents an increase caused by prednisone; down arrow represents a decrease caused by prednisone. Pred, prednisone. *N* = 5/group.

Simultaneously, the effects of prednisone on the gut microbiota were also different in the three types of MRL/lpr mice. Prednisone imposed no effects on alpha diversity (Chao1, Shannon) in control mice, decreased the Chao1 index in the control and early and short-term antibiotic exposure mice, and increased alpha diversity (Chao1, Shannon) in the early and short-term FMT-treated mice (Figures 5A,B). LEfSe analysis was performed to reveal microbial genera that were altered by prednisone in the three types of lupus mice (Supplementary Figures S2–S4). A Venn diagram showed the shared or unique altered genera of the gut microbiota in the three types of lupus mice. As shown in Figure 5C, a total of 6, 11, and 14 genera altered by prednisone were observed in control, antibiotic- and FMT-treated mice, respectively. Only two altered genera were shared by the three types of lupus mice: prednisone increased the abundance of (*Prevotella*) and *Parabacteroides* in control and antibiotic-treated mice but decreased these bacteria in FMT mice (Figure 5C). Additionally, the increase in *Prevotella* caused by prednisone was shared in control and antibiotic-treated mice. Five genera altered by prednisone were also shared in antibiotic- and FMT-treated mice but the effects of prednisone on the four shared altered genera (*Dehalobacterium*, *Dorea*, *Sutterella*, and *Turicibacter*) were different. No shared altered genera were observed in control and FMT-treated mice (Figure 5C). The uniquely altered genera in control mice were *Proteus*, *Bilophila*, and *Klebsiella*, which were all decreased by prednisone. The uniquely altered genera in antibiotic-treated mice were *Allobaculum*, *Bifidobacterium*, and *Adlercreuzia*, which were also all decreased by prednisone. Seven genera were uniquely altered by prednisone in FMT-treated mice, including decreased *Bacteroides* and *Akkermansia* and increased (*Ruminococcus*), *Odoribacter*, *Coprococcus*, *Ruminococcus*, and *Desulfovibrio* (Figure 5C).

## Discussion

The gut microbiota was strongly correlated with SLE, but its role in affecting SLE was not clear. Hence, an increasing number of studies have investigated the roles of the gut microbiota in SLE by intervening gut microbiota. Some commensal bacteria, including *Lactobacillus* sp. (Mu et al., 2017b), *Enterococcus gallinarum* (Manfredo Vieira et al., 2018), and *Ruminococcus gnavus* (Azzouz et al., 2019), have potential to control lupus activity in mice. Using the MRL/lpr murine model, this study had proven that early and short-term interventions in the gut microbiota could affect lupus severity, progression, and treatment.

The causative mutation, Fas^lpr^, promoted the survival of self-reactive lymphocytes, leading to immune proliferation, lymphadenopathy, the emergence of anti-DNA antibodies, and fatal immune complex glomerulonephritis in MRL/lpr mice (Schile et al., 2018). This study demonstrated that the early and short-term interventions in the gut microbiota could affect lupus-like conditions caused by genetic mutations. At the early stage, the depletion of microbiota through antibiotics exposures resulted in increased lupus severity in MRL/lpr mice. This finding was inconsistent with the Mu et al. (2017a) study showing that antibiotics ameliorate lupus-like symptoms in mice. The reasons for this inconsistence might be as follows: (i) oral antibiotics were given at the initial 2 weeks in this study but were given over the course of 8 weeks in the Mu’s study, since the length of antibiotics use could cause the different effects of antibiotics on gut microbiota (Wei et al., 2018); (ii) The dose of antibiotics used in the Mu’s study was five times that in this study, but different doses of antibiotic had a differing impacts on gut microbiota (Lange et al., 2016); (iii) oral antibiotics were given before onset in this study but were given after onset in the Mu’s study. Therefore, the antibiotics-induced alterations in the gut microbiota and lupus activity were different from the Mu’s study. In this study, some altered genera caused by early and short-term antibiotics exposure were associated with increased lupus severity. Some genera reduced by antibiotics exposure, including *Lactobacillus* and *Bifidobacterium*, were reported to have the ability to alleviate lupus activity (Esmaeili et al., 2017; Mu et al., 2017b). In addition, increased levels of *Klebsiella* and *Proteus* caused by antibiotics exposure could induce increased lupus severity. Both *Klebsiella* and *Proteus* were found to be the infectious bacteria in SLE patients (Fei et al., 2014; Chen and Zhan, 2017). Not only that, the antibodies to *Klebsiella* were positively correlated with anti-dsDNA antibodies in SLE patients (Harkiss et al., 1988). The genus *Proteus* was not reported to trigger lupus, but it was the main microbial culprit in the causation of rheumatoid arthritis through *Proteus* peptide (Christopoulos et al., 2017). The early and short-term interventions in the gut microbiota affected not only lupus severity but also lupus progression in MRL/lpr mice. Genetic factors caused increased lupus severity from 9 to 13 weeks old in the control MRL/lpr mice (Liu et al., 2006). The early and short-term antibiotics exposure imposed no effects on lupus progression but FMT suppressed lupus progression. This observation indicated that temporal changes in the gut microbiota are related to disease progression, which is in agreement with the previous study (Schirmer et al., 2018). In sum, the short-term interventions in the gut microbiota impose long-lasting effects on lupus severity.

Not only that, the short-term interventions in the gut microbiota before onset also affected lupus treatment of prednisone. Both short-term treatments by antibiotics and FMT before onset inhibited the therapeutic efficiency of prednisone in MRL/lpr mice. In the control MRL/lpr mice, prednisone had obvious therapeutic effects on lupus disease, which might be due to the downregulation of *Proteus*, *Klebsiella*, and *Bilophila* caused by prednisone. As mentioned above, *Proteus* and *Klebsiella* showed the potential to aggravate lupus conditions (Harkiss et al., 1988). *Bilophila* could cause a systemic inflammatory response in specific-pathogen-free mice (Feng et al., 2017). In the early and short-term antibiotics exposure mice, the weaken therapeutic efficiency of prednisone might be due to the decreases in the abundance of *Allobaculum*, *Bifidobacterium*, and *Adlercreutzia*, which were all negatively correlated with lupus activity and reported to be capable of immunoregulatory in the intestines (Schiavi et al., 2016; Calvo-Barreiro et al., 2018; Scott et al., 2018). In the early and short-term FMT-treated mice, low lupus activity might explain no significant effects of prednisone for lupus. Disease activity has a big impact on the effectiveness of treatment for lupus (Pego-Reigosa et al., 2015). This study demonstrated that the short-term changes of gut microbiota before onset affected not only therapeutic efficiency of prednisone but also the targeted gut microbiota of prednisone. Therefore, gut microbiota could play a direct role in treating SLE or an auxiliary role in improving the efficiency of drugs on lupus.

This study successfully demonstrated that the short-term intervention in the gut microbiota before lupus onset could affect lupus severity, progression and treatment. Gut microbiota could be an important reference for choosing therapeutic schedules in the clinical treatment of SLE. However, this study did not validate the results through human samples and reveal the relevant mechanism from the perspective of the host. Even so, this study is the first to reveal the gut microbiota as an important factor of therapeutic efficiency of glucocorticoids on lupus.

## Data Availability Statement

The raw sequences of gut microbiota have been submitted to NCBI Project under accession number PRJNA532822 with NCBI Sequence Read Archive under accession number SRP192681.

## Ethics Statement

All animal handling and experimental procedures were performed in accordance with local ethical committees and the National Institutes of Health Guide for the Care and Use of Laboratory Animals. All efforts were made to minimize animal suffering and to reduce the number of animals used. All procedures performed in this study involving animals were approved by the Ethics Committee of Zhejiang Chinese Medical University.

## Author Contributions

ZH and CW conceptualized and designed the study. YZ, QL, YY, and MW were responsible for the acquisition of the data. ZH and YZ worked on the analysis, interpretation of data and drafting of the manuscript. All authors approved the final manuscript.

## Conflict of Interest

The authors declare that the research was conducted in the absence of any commercial or financial relationships that could be construed as a potential conflict of interest.

## Footnotes

1 http://huttenhower.sph.harvard.edu/lefse/

## Abbreviations

SLE: systemic lupus erythematosus
FMT: fecal microbiota transplantation
Th17: T-helper 17 cells
Treg: regulatory T cells
QIIME: quantitative insights into microbial ecology
OTUs: operational taxonomical units
LEfSe: linear discriminant analysis effect size
LDA: linear discriminant analysis
PCoA: principal coordinates analysis
Cr: creatinine
ALT: alanine aminotransferase
LDH: lactate dehydrogenase
dsDNA: double-stranded DNA
IFN- α: interferon- α
IL-6: interleukin 6
sIgA: secretory immunoglobulin A.
