# Supplementary material for: Early and Short-Term Interventions in the Gut Microbiota Affects Lupus Severity, Progression, and Treatment in MRL/lpr Mice
Source: Front Microbiol. 2020 Apr 14;11:628. doi: 10.3389/fmicb.2020.00628 (PMC7171286; doi:10.3389/fmicb.2020.00628)
Supplement: Supplementary file 1 [file Data_Sheet_1.docx]

**Early and short-term interventions in the Gut Microbiota Affects Lupus Severity, Progression and Treatment in MRL/lpr Mice**

Yun Zhang^#^, Qiuping Liu^#^, Yiran Yu, Mingzhu Wang, Chengping Wen* and Zhixing He* ^[[1]](#footnote-1)^

Institute of Basic Research in Clinical Medicine, College of Basic Medical Science, Zhejiang Chinese Medical University, Hangzhou 310053, China.

*Corresponding author. Address: Institute of Basic Research in Clinical Medicine, College of Basic Medical Science, Zhejiang Chinese Medical University, Hangzhou 310053, China.

“#”: These authors contributed equally to this work.

Tel.: 086-571-86613587.

E-mail address: wengcp@yeah.net (Chengping Wen), hzx52871069@yeah.net (Zhixing He)

**Summary of supporting information:**

**A. SUPPORTING FIGURES S1-S4**

**Figure S1** LEfSe identified the differential microbial taxa between NC and MT (A)，MT and AT (B), between AT and FMT (C), and between MT and FMT (D). Significant differences are shown (LDA score >2). NC: The C57/BL6 mice treated with PBS solution from 6 weeks old to 9 weeks old; MT: the MRL/lpr mice treated with PBS solution from 6 weeks old to 9 weeks old; AT: the MRL/lpr mice treated with antibiotics from 6 weeks old to 8 weeks old and PBS solution from 8 weeks old to 9 weeks old; FMT: the MRL/lpr mice treated with antibiotics from 6 weeks old to 8 weeks old and fecal microbiota transplantation from 8 weeks old to 9 weeks old.

**Figure S2** Prednisone caused the alterations in gut microbiota at 5w (A) and 7w (B) in the control MRL/lpr mice, which were identified by LEfSe method. Significant differences are shown (LDA score >2).

**Figure S3** Prednisone caused the alterations in gut microbiota at 5w (A) and 7w (B) in the antibiotics-treated mice, which were identified by LEfSe method. Significant differences are shown (LDA score >2).

**Figure S4** Prednisone caused the alterations in gut microbiota at 5w (A) and 7w (B) in the fecal microbiota transplantation-treated mice, which were identified by LEfSe method. Significant differences are shown (LDA score >2).

**
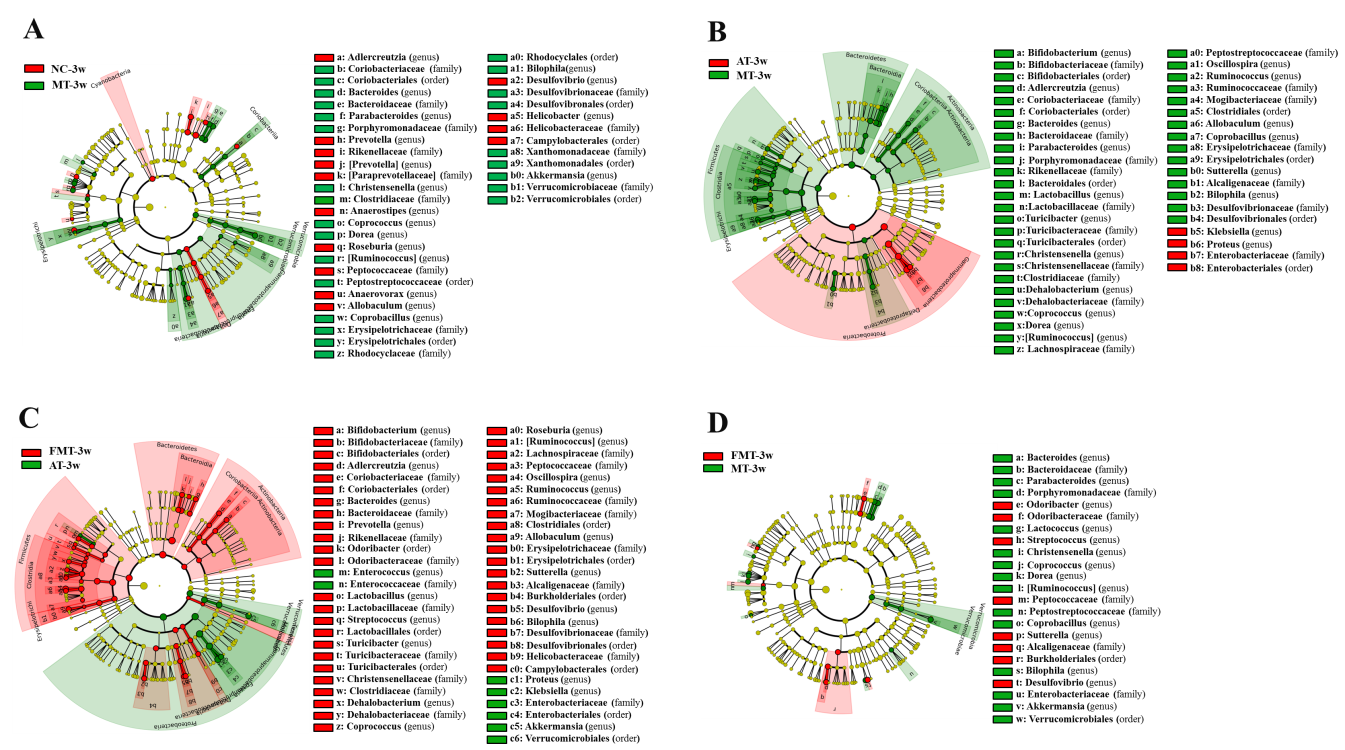
**

**Figure S1** LEfSe identified the differential microbial taxa between NC and MT (A)，MT and AT (B), between AT and FMT (C), and between MT and FMT (D). Significant differences are shown (LDA score >2). NC: The C57/BL6 mice treated with PBS solution from 6 weeks old to 9 weeks old; MT: the MRL/lpr mice treated with PBS solution from 6 weeks old to 9 weeks old; AT: the MRL/lpr mice treated with antibiotics from 6 weeks old to 8 weeks old and PBS solution from 8 weeks old to 9 weeks old; FMT: the MRL/lpr mice treated with antibiotics from 6 weeks old to 8 weeks old and fecal microbiota transplantation from 8 weeks old to 9 weeks old.

**
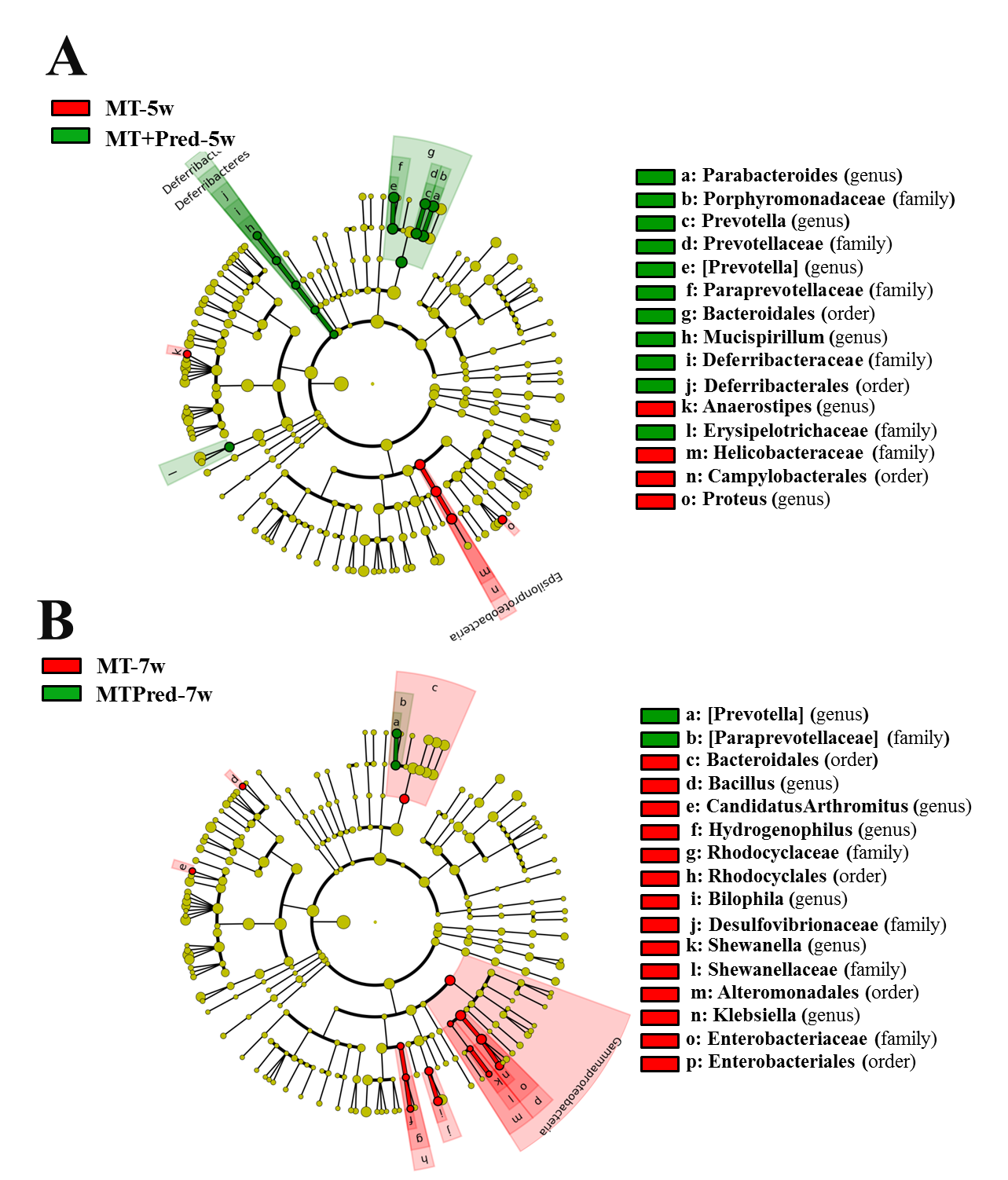
**

**Figure S2** Prednisone caused the alterations in gut microbiota at 5w (A) and 7w (B) in the control MRL/lpr mice, which were identified by LEfSe method. Significant differences are shown (LDA score >2).

**
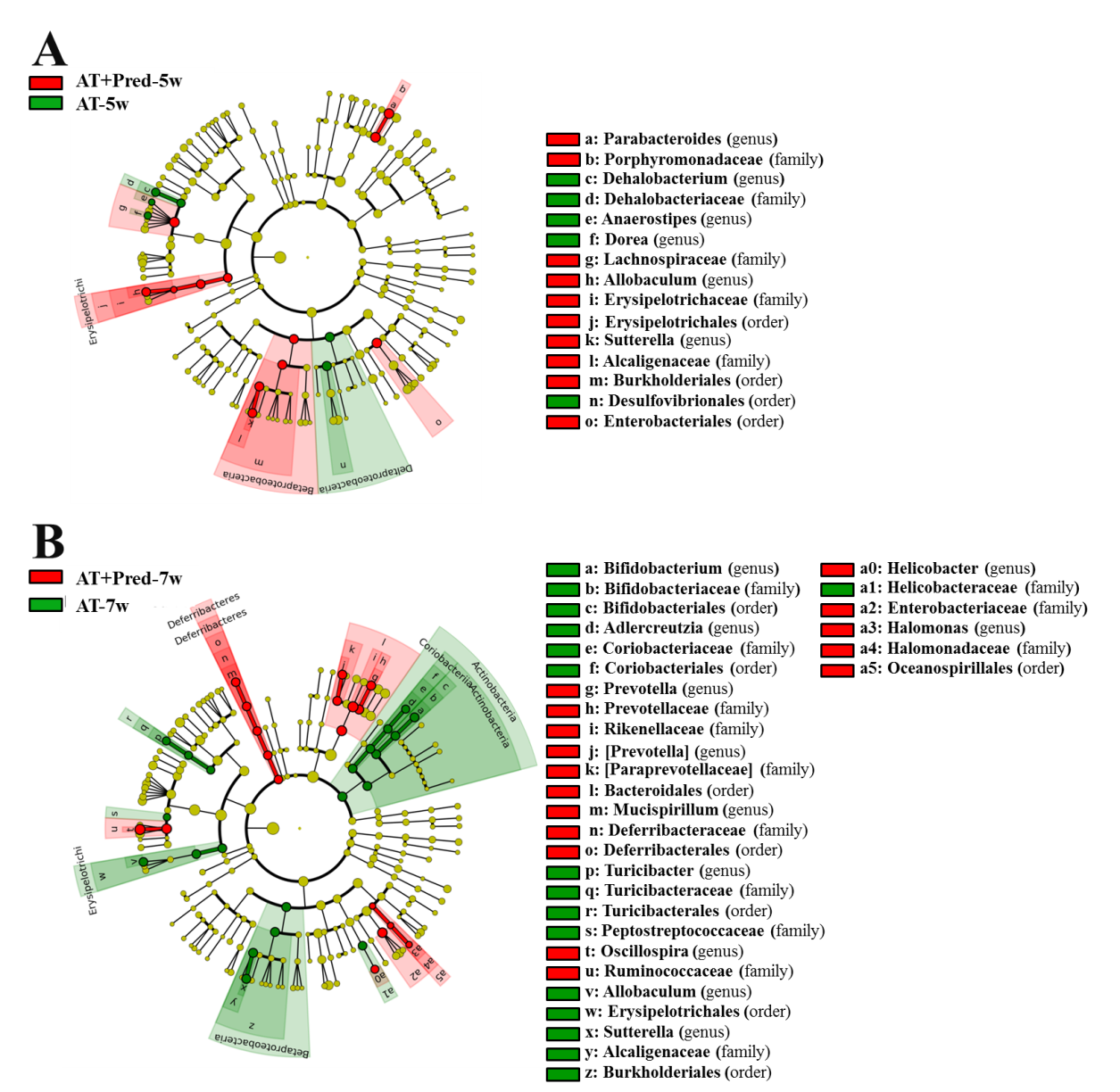
**

**Figure S3** Prednisone caused the alterations in gut microbiota at 5w (A) and 7w (B) in the antibiotics-treated mice, which were identified by LEfSe method. Significant differences are shown (LDA score >2).

**
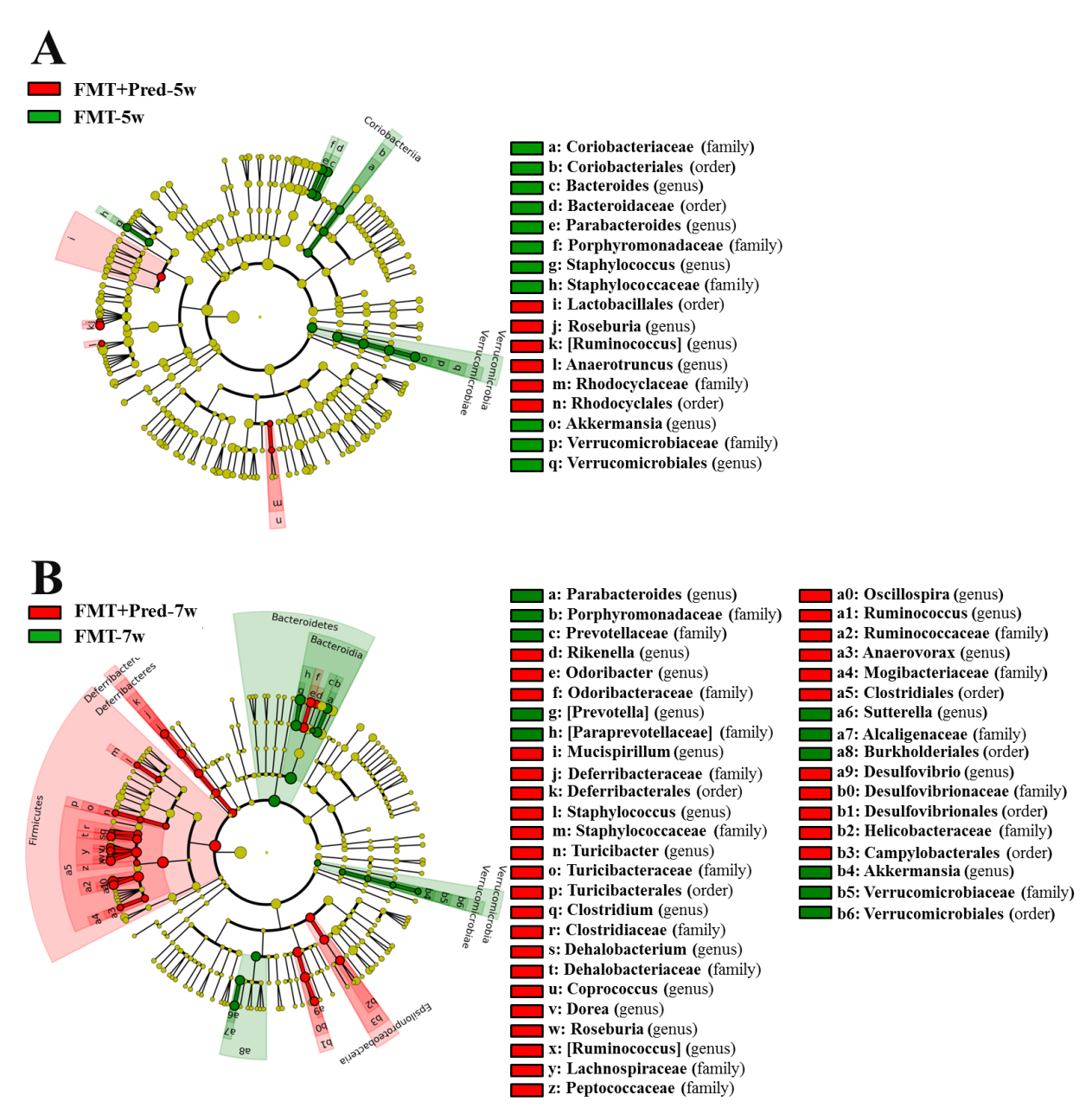
**

**Figure S4** Prednisone caused the alterations in gut microbiota at 5w (A) and 7w (B) in the fecal microbiota transplantation-treated mice, which were identified by LEfSe method. Significant differences are shown (LDA score >2).

1. *Corresponding author. Address: Institute of Basic Research in Clinical Medicine, College of Basic Medical Science, Zhejiang Chinese Medical University, Hangzhou 310053, China.

   “+”: These authors contributed equally to this work.

   Tel.: 086-571-86613587.

   E-mail address: wengcp@163.com (Chengping Wen), hzx2015@zcmu.edu.cn (Zhixing He) [↑](#footnote-ref-1)
